# Effects of CB2 Receptor Modulation on Macrophage Polarization in Pediatric Inflammatory Bowel Disease

**DOI:** 10.3390/ijms26083720

**Published:** 2025-04-15

**Authors:** Mara Creoli, Alessandra Di Paola, Antonietta Tarallo, Sohail Aziz, Erasmo Miele, Massimo Martinelli, Marianna Casertano, Antonio Colucci, Sabrina Cenni, Maria Maddalena Marrapodi, Annamaria Staiano, Francesca Rossi, Caterina Strisciuglio

**Affiliations:** 1 Department of Woman, Child and General and Specialist Surgery, University of Campania “Luigi Vanvitelli”, Via Luigi de Crecchio 2, 80138 Naples, Italy; mara.creoli@unicampania.it (M.C.); alessandra.dipaola@unicampania.it (A.D.P.); antonio.colucci2209@gmail.com (A.C.); mariamaddalena.marrapodi@unicampania.it (M.M.M.); caterina.strisciuglio@unicampania.it (C.S.); 2 Department of Life Sciences, Health and Health Professions, Link Campus University, Via del Casale di San Pio V, 00165 Rome, Italy; 3 Department of Translational Medical Science, Section of Pediatrics, University of Naples “Federico II”, Via S. Pansini 5, 80131 Naples, Italy; antonietta.tarallo@unina.it (A.T.); erasmo.miele@unina.it (E.M.); massimo.martinelli@unina.it (M.M.); mariannacasertano@gmail.com (M.C.); staiano@unina.it (A.S.); 4 Department of Experimental Medicine, University of Campania “Luigi Vanvitelli”, Via Santa Maria di Costantinopoli 16, 80138 Naples, Italy; sohail.aziz@studenti.unicampania.it (S.A.); sabrinacenni3@gmail.com (S.C.)

**Keywords:** inflammatory bowel disease, M1, M2, macrophage polarization, Caco-2 cells, intestinal barrier, CB2 receptor, Crohn’s disease, ulcerative colitis

## Abstract

Macrophages play a crucial role in maintaining intestinal homeostasis and can exhibit either pro-inflammatory M1 or anti-inflammatory M2 phenotypes. The cannabinoid receptor type 2 (CB2) is involved in immune regulation and may represent a therapeutic target in inflammatory bowel disease (IBD). Our study investigates the phenotype of circulating macrophages and CB2 expression in children with IBD, assessing the role of CB2 stimulation in macrophage polarization, iron metabolism, and intestinal barrier function. Macrophages were isolated from 17 children with ulcerative colitis (UC), 21 with Crohn’s disease (CD), and 12 healthy controls (CTR). Cells were treated with a CB2 agonist (JWH-133) and an inverse agonist (AM630). CB2 expression and macrophage polarization were assessed by Western blot. Iron metabolism was evaluated through IL-6, hepcidin levels, FPN-1 expression, and iron concentration. Inflammation was assessed by cytokine release. An in vitro “immunocompetent gut” model was used to study the effects of CB2 stimulation on macrophage polarization and intestinal barrier function. CB2 expression was reduced in IBD macrophages. Compared to controls, IBD patients showed increased M1 markers and pro-inflammatory cytokines, with a reduction in M2 markers and IL-13. Altered iron metabolism was observed, with increased [Fe^3+^], hepcidin release, and DMT1 expression, and reduced FPN-1. CB2 stimulation restored iron metabolism, induced M2 polarization, and improved intestinal barrier function. CB2 could represent a novel therapeutic target for IBD by modulating macrophage function, iron metabolism, and mucosal barrier restoration.

## 1. Introduction

Chronic gastrointestinal inflammation is a hallmark of the multifaceted and complex group of disorders known as inflammatory bowel disease (IBD) [1]. In pediatric patients, IBD can significantly affect growth, development, and overall quality of life, underscoring the importance of understanding its underlying pathogenesis and developing novel treatment strategies [2]. IBD is characterized by a dysregulated immune response, involving complex interactions among several immune cells, cytokines, as well as inflammatory mediators [3]. Macrophages play a key role in this complex immunological milieu, demonstrating exceptional adaptability and versatility in coordinating inflammatory and anti-inflammatory responses [4]. Depending on external stimuli and signaling pathways, macrophages in the inflamed gut may take on different functional phenotypes that can be categorized broadly as pro-inflammatory (M1) or anti-inflammatory (M2) [5]. M1 macrophages exert pro-inflammatory properties, releasing pro-inflammatory chemokines and cytokines. M2 macrophages exhibit anti-inflammatory activities, by producing anti-inflammatory cytokines, involved in tissue remodeling and post-injury repair, and promoting angiogenesis [5,6]. The endocannabinoid system, in particular the cannabinoid receptor 2 (CB2), is widely recognized for its potent anti-inflammatory and immunoregulatory properties, playing a key role in modulating immune cell function and suppressing pro-inflammatory cytokine production and, in recent years, targeting CB2 has garnered increasing attention as a possible therapeutic avenue [7]. The CB2 receptor is mainly expressed in immune cells, such as macrophages and lymphocytes, and it has shown promise as a therapeutic target in the treatment of inflammatory disorders, including IBD. It has already been reported that there is an important anti-inflammatory role of the CB2 receptor in several inflammatory and autoimmune diseases. In particular, we have previously demonstrated that CB2 acts as an immunosuppressive and anti-inflammatory mediator in pediatric immune thrombocytopenia, by restoring the function and viability of mesenchymal stromal cells [8]. Moreover, CB2 stimulation also reduces the obesity-related inflammatory state in obese-derived adipocytes [9,10].

Recently, we also described an important anti-inflammatory role of the CB2 receptor in containing inflammation and in promoting the macrophages’ switch toward the anti-inflammatory M2 phenotype in Duchenne disease [11].

Previous studies have highlighted the involvement of the CB2 receptor also in IBD [7,12,13]. Notably, the CB2 Q63R variant (rs35761398), a polymorphism in the CB2-encoding gene, has been associated with a less functional isoform of the receptor, leading to an increased risk of inflammatory and autoimmune diseases [14]. Our earlier findings demonstrated a significant association between the CNR2 rs35761398 polymorphism and susceptibility to pediatric IBD, particularly Crohn’s disease, as well as a more severe clinical phenotype in both UC and CD [14]. Furthermore, we have identified a critical role of the CB2 receptor in bone metabolism, where its activation exerts protective, anti-osteoporotic effects. Specifically, our research demonstrated that CB2 stimulation counteracts IBD-related osteoporosis by directly modulating osteoclast activity and indirectly influencing iron metabolism [15].

Stimulation of CB2 receptors promotes a macrophage phenotype shift toward the anti-inflammatory M2 type, thereby reducing inflammation and mitigating epithelial dysfunction [16]. Nevertheless, understanding the exact impact of CB2 receptor modulation on macrophage polarization, specifically in pediatric IBD, needs further research and discussion. In our recent studies on celiac disease, we provided evidence supporting CB2 receptor involvement in disease pathogenesis, reinforcing its potential as a key target for anti-inflammatory and immunomodulatory therapies [15,17,18]. Therefore, building on these findings and the intriguing properties of CB2 in pediatric celiac disease, we decided to conduct a parallel study on pediatric IBD, using the same approach while taking into consideration the unique features of these diseases [18]. To this end, we isolated macrophages from the peripheral blood of pediatric patients with Crohn’s disease (CD) and ulcerative colitis (UC) to assess CB2 receptor expression and macrophage phenotypes, and to evaluate the impact of CB2 pharmacological modulation on macrophage polarization in both conditions. We also generated an in vitro model of the “immunocompetent gut” [19], constituted by CD and UC macrophages and the human epithelial cell line, Caco-2. This model was employed to explore the role of CD and UC macrophages in compromising intestinal barrier integrity and to assess whether modulating macrophage polarization could promote its restoration.

## 2. Results

### 2.1. Characterization of IBD Macrophages

**Figure 1:**
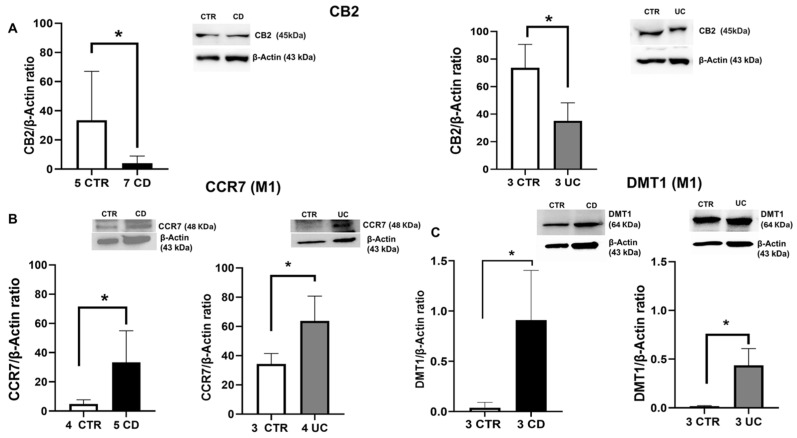
Characterization of CD and UC macrophages. CB2 (**A**), CCR7 (**B**), and DMT1 (**C**) protein expression, determined by Western blot loading 15 μg of total lysate, in macrophages from Crohn’s disease (CD) and ulcerative colitis (UC) patients compared with macrophages from healthy controls (CTR). The most representative images are displayed. The protein bands were detected through Image Studio Digits software and Image Lab Ink software “BIORAD”. Band intensity ratios were quantified relative to CTR samples after normalization to respective controls. Histograms display the relative quantification of CB2, CCR7, and DMT1 expression levels, normalized to the housekeeping protein β-Actin, and presented as mean ± SD from independent experiments on individual samples. Statistical analysis was performed using a *t*-test. * Indicates *p* ≤ 0.05 versus CTR.

We assessed CB2 protein expression levels in macrophages isolated from the peripheral blood of UC and CD patients using Western blot analysis. Our findings revealed that CB2 protein expression levels were significantly lower in UC and CD macrophages compared to CTR macrophages, allowing us to confirm its involvement in these other inflammatory diseases (Figure 1A).

**Figure 2:**
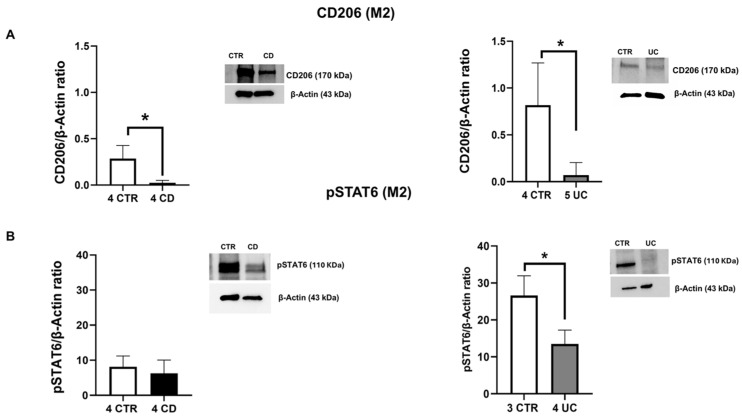
Characterization of CD and UC macrophages. CD206 (**A**), pSTAT6 (**B**) protein expressions, determined by Western blot loading 15 μg of total lysate, in macrophages from Crohn’s disease (CD) and ulcerative colitis (UC) patients compared with macrophages from healthy controls (CTR). The most representative images are displayed. The protein bands were detected through Image Lab Ink software “BIORAD”. Band intensity ratios of immunoblots relative to CTR were quantified after normalization to respective controls. Histograms show the relative quantification of CD206 and pSTAT6 expression levels, normalized to the housekeeping protein β-Actin, and are presented as mean ± SD from independent experiments on individual samples. Statistical analysis was performed using a *t*-test. * Indicates *p* ≤ 0.05 versus CTR.

Furthermore, to investigate the phenotype of IBD macrophages, we evaluated the protein expression levels of M1 polarization markers, CCR7 and DMT1 (Figure 1B,C), and M2 polarization markers, CD206 and pSTAT6 (Figure 2A,B). In particular, we observed significantly high levels of both CCR7 and DMT1, together with a significant reduction in the M2 phenotype marker, CD206, and a decreasing trend in the phenotype switching marker pSTAT6 in IBD macrophages compared to CTR macrophages. Statistically significant results were noted for CCR7 and DMT1 expression in CD macrophages, for CD206 levels in both IBD macrophages, and for pSTAT6 expression in macrophages derived by UC patients. These results confirm the predominance of the M1 macrophage inflammatory phenotype in IBD patients.

**Figure 3:**
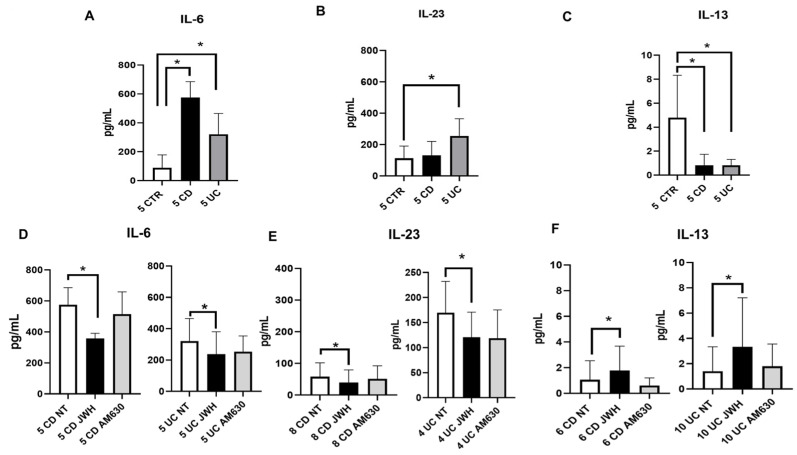
Cytokine concentration evaluation in CD and UC macrophages before and after CB2 modulation. IL-6 (**A**), IL-23 (**B**), and IL-13 (**C**) concentrations (pg/mL) in macrophages from Crohn’s disease (CD) and ulcerative colitis (UC) patients compared with macrophages from CTR, determined by ELISA assay. IL-6 (**D**), IL-23 (**E**), and IL-13 (**F**) concentrations (pg/mL) in macrophages from CD and UC patients after 48 h JWH-133 [100 nM] and AM630 [10 µM] treatment, determined by ELISA assay. Histogram shows cytokines concentration expressed as mean ± SD from independent experiments on individual samples of IL-6, IL-23, and IL-13 concentrations were determined using standard calibration curves. Statistical analysis was performed using a *t*-test. * Indicates *p* ≤ 0.05 versus CTR or NT.

Using enzyme-linked immunosorbent assays (ELISA), we also investigated cytokines release by CD and UC macrophages compared to CTR macrophages (Figure 3).

Our analysis revealed a statistically significant increase in the level of pro-inflammatory cytokines IL-6 in both UC and CD macrophages and IL-23 in UC (Figure 3A,B). In addition, we found a statistically significant decrease in the anti-inflammatory cytokine IL-13 in macrophages derived from both pathologies (Figure 3C). These data confirm the presence of an altered inflammatory status in IBD patients.

### 2.2. Effects of CB2 Modulation on IBD Macrophage Polarization and Inflammatory Profile

**Figure 4:**
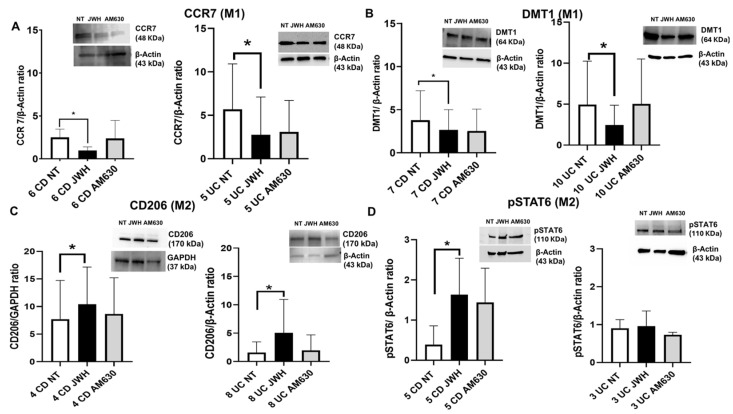
Effects of CB2 modulation on CD and UC macrophage polarization. CCR7 (**A**), DMT1 (**B**) CD206 (**C**), and pSTAT6 (**D**) protein expressions, determined by Western blot, loading 15 μg of total lysate, in macrophages from Crohn’s disease (CD) and ulcerative colitis (UC) patients after JWH-133 [100 nM] and AM630 [10 µM] treatment for 48 h. The most representative images are displayed. Image Studio Digits software has been used to detect protein bands. The protein bands were detected through Image Studio Digits software and Image Lab Ink software “BIORAD”. Band intensity ratios of immunoblots relative to non-treated (NT) samples were quantified after normalization to respective controls. Histograms display the relative quantification of CCR7, DMT1, CD206, and pSTAT6 expression levels, normalized to the housekeeping proteins GAPDH (**C**) or β-Actin (**A**–**D**), and are presented as mean ± SD from independent experiments on individual samples. Statistical analysis was performed using a *t*-test. * Indicates *p* ≤ 0.05 versus NT.

We examined the effect of CB2 receptor stimulation on IBD macrophage polarization and cytokine release by treating macrophages isolated from IBD patients with the CB2 selective agonist JWH-133 [100 nM] and the CB2 inverse agonist AM630 [10 µM]. Following 48 h of drug incubation, we analyzed the effects of JWH-133 and AM630 on macrophages polarization with Western blotting, as well as on inflammatory profile by evaluating pro- and anti-inflammatory cytokines levels through ELISA. JWH-133 treatment resulted in a statistically significant reduction in M1 markers CCR7 and DMT1 in both UC and CD macrophages (Figure 4A,B). Furthermore, following JWH-133 treatment, we observed a statistically significant increase in CD206 protein expression levels in both UC and CD macrophages (Figure 4C), along with an upregulation of pSTAT6 observed exclusively in CD macrophages (Figure 4D). There was a trend towards increased pSTAT6 expression in UC macrophages, even though not in a statistically significant manner (Figure 4D). These findings suggest that CB2 stimulation can induce macrophage polarization toward the anti-inflammatory M2 phenotype.

As expected, AM630 [10 µM] exposure induced an effect opposite to that observed with JWH-133 stimulation. ELISA assays revealed a significant reduction in IL-6 and IL-23 levels in both CD and UC macrophages (Figure 3D,E), along with a significant increase in the anti-inflammatory cytokine IL-13 following JWH-133 stimulation (Figure 3F), confirming Western blotting results. Conversely, AM630 administration increased the release of IL-6 and IL-23 in both pathologies. These obtained results confirmed the potential anti-inflammatory role of CB2 also in IBD macrophages.

### 2.3. Evaluation of Iron Metabolism in IBD Macrophages

**Figure 5:**
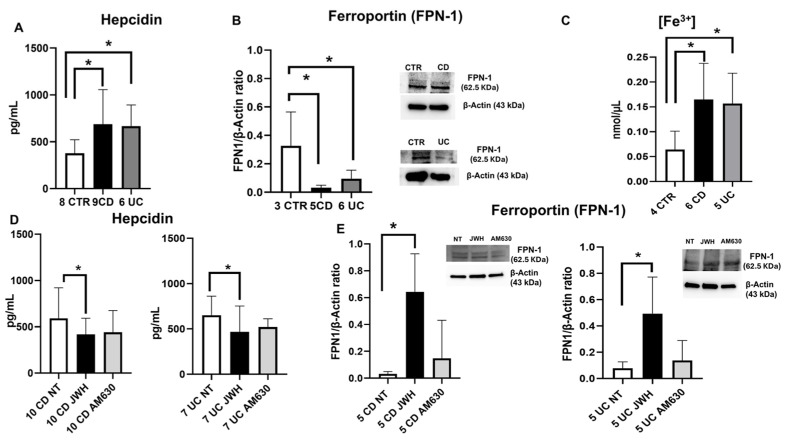
Iron metabolism evaluation in CD and UC macrophages before and after CB2 modulation. Hepcidin concentrations (pg/mL) (**A**) in macrophages from Crohn’s disease (CD) and ulcerative colitis (UC) patients compared with macrophages from healthy donors (CTR), and (**D**) in macrophages from CD and UC patients after 48 h JWH-133 [100 nM] and AM630 [10 µM] treatment, determined by ELISA assay. The cytokine concentration was analyzed on a standard concentration curve according to the manufacturer’s instructions. Histogram shows hepcidin concentration as the mean ± SD of independent experiments on each individual sample. Ferroportin (FPN-1) protein expression, determined by Western blot, loading 15 μg of total lysate, (**B**) in macrophages from CD and UC patients compared with macrophages from CTR, and (**E**) in macrophages from CD and UC patients after JWH-133 [100 nM] and AM630 [10 µM] treatment for 48 h. The most representative images are displayed. The protein bands were detected through Image Lab Ink software “BIORAD”. Band intensity ratios of immunoblots, relative to CTR or non-treated (NT) samples, were quantified after normalization to respective controls. Histograms display the relative quantification of FPN-1 expression levels, normalized to the housekeeping protein β-Actin, and presented as mean ± SD from independent experiments on individual samples. Fe^3+^ intracellular concentrations (nmol/µL) (**C**) in macrophages from CD and UC patients compared to CTR were determined by Iron Assay. Histograms show Fe^3+^ concentrations expressed as mean ± from independent experiments on individual samples. The most representative images are displayed. The protein bands were detected through Image Lab Ink software “BIORAD”. Statistical analysis was performed using a *t*-test. * Indicates *p* ≤ 0.05 versus CTR or NT.

To explore iron metabolism in IBD macrophages, we assessed hepcidin levels by ELISA, FPN-1 protein expression levels by WB, and intracellular ferric iron concentration [Fe^3+^] by Iron Assay (Figure 5). Our results showed a significant increase in hepcidin levels in both diseases (Figure 5A), with a consequent reduction in FPN-1 expression (Figure 5B). These outcomes align with the significantly high levels of IL-6 released by IBD macrophages (Figure 3A), an important mediator of hepcidin upregulation [20]. Moreover, in line with these results, we observed a statistically significant rise in intracellular iron concentration (Fe^3+^) in IBD macrophages, compared to CTR (Figure 5C). These data are also supported by the statistically significant increase in the iron importer DMT1 observed in both CD and UC macrophages compared to CTR (Figure 1C), which contributes to iron internalization and increases its intracellular concentration, thereby worsening the inflammatory state in IBD macrophages.

### 2.4. Effects of CB2 Modulation on IBD Macrophages’ Iron Metabolism

To elucidate the impact of CB2 receptor stimulation on IBD macrophages’ iron metabolism, we evaluated the effects of JWH-133 [100 nM] and AM630 [10 µM] administration after 48 h of incubation on hepcidin release, and FPN-1 protein expression via ELISA assays and WB, respectively (Figure 5). After JWH-133 treatment, we observed a significant reduction in hepcidin concentration in both UC and CD macrophages, whereas no notable changes in hepcidin release were observed following stimulation with the inverse agonist AM630 (Figure 5D). Additionally, we noticed a statistically significant increase in FPN-1 expression levels after JWH-133 administration in macrophages derived from IBD patients compared to NT; while, as expected, AM630 induced the opposite effect on FPN-1 levels (Figure 5E).

### 2.5. Effect of CB2 Modulation on IL-6 and IL-23 Release by Caco-2 Cells Alone and in Co-Culture with IBD Macrophages

We investigated the effects of the pharmacological modulation of CB2 receptors on intestinal barrier functionality, generating an in vitro model of the intestinal epithelial barrier, and co-culturing IBD macrophages with Caco-2 cells.

**Figure 6:**
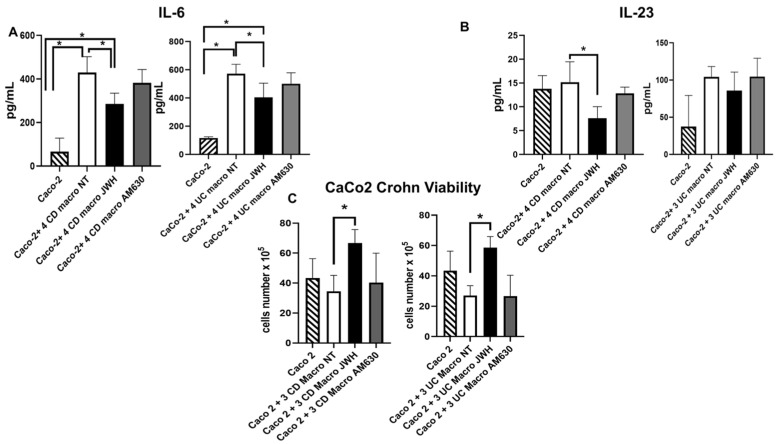
Effects of CB2 modulation on cytokine release by Caco-2 cells and on Caco-2 viability (**A**–**C**). IL-6 and IL-23 concentrations (pg/mL) were measured by ELISA in Caco-2 cells cultured alone and in coculture with macrophages from Crohn’s disease (CD) and ulcerative colitis (UC) patients, after 48 h treatment with JWH-133 [100 nM] or AM630 [10 µM]. Cytokine concentrations were determined using standard calibration curves according to the manufacturer’s instructions. Histograms show cytokine concentrations expressed as mean ± SD from independent experiments on individual samples. The cytokine concentration was analyzed on a standard concentration curve according to the manufacturer’s instructions. Caco-2 cell viability (**C**) was assessed by cytofluorimetric assay after coculture with CD and UC macrophages and 48 h treatment with JWH-133 or AM630. Statistical analysis was performed using a *t*-test and one-way ANOVA. * Indicates *p* ≤ 0.05 versus Caco-2 + CD or UC macrophages NT.

We showed an increase in pro-inflammatory cytokine levels released by Caco-2 cells co-cultured with untreated IBD macrophages compared to Caco-2 cells alone. Following JWH-133 treatment, IL-6 levels were significantly reduced in both CD and UC conditions compared to untreated co-cultured Caco-2 cells and Caco-2 cells alone. Additionally, IL-23 levels were also reduced, with a significant decrease observed in CD compared to untreated co-cultured Caco-2 cells. Conversely, AM630 induced an increase in pro-inflammatory cytokine release by Caco-2 cells compared to Caco-2 cells alone, particularly for IL-6 in both conditions and for IL-23 in CD, while a trend toward increased IL-23 levels was observed in UC, suggesting a worsening of the basal condition (Figure 6A,B).

### 2.6. Effect of CB2 Modulation on Caco-2 Viability Alone and in Co-Culture with CD and UC Macrophages

Finally, we evaluated the viability of Caco-2 cells, both alone and in co-culture with IBD macrophages, treated or untreated with JWH-133 [100 nM] and AM630 [10 µM] for 48 h (Figure 6C). Co-cultured with CD and UC, M1 macrophages lead to a decrease in Caco-2 cell viability, supporting our hypothesis that IBD M1 macrophages contribute to epithelial damage. Following JWH-133 treatment, we observed a restoration of Caco-2 viability, with a significant increase in the number of viable Caco-2 cells. Conversely, the inverse agonist AM630 produced the expected opposite effect, reducing the number of viable cells to a level comparable to that observed when Caco-2 cells were co-cultured with CD and UC M1 macrophages.

## 3. Discussion

Inflammatory bowel disease (IBD), a complex group of disorders due to chronic gastrointestinal inflammation, is characterized by immune response alteration, including impairment of several immune cell activities and functions [3]. Recent research has underscored the pivotal role of macrophages in maintaining intestinal homeostasis and their involvement in the inflammation associated with IBD. Therefore, emerging therapeutic approaches for IBD are increasingly focusing on promoting macrophage polarization towards an anti-inflammatory M2 phenotype, with the aim of counteracting the chronic inflammatory immune response that typifies the disease [21,22,23,24].

In line with this therapeutic perspective, our recent work demonstrated that CB2 stimulation promotes the polarization of macrophages from a pro-inflammatory M1 phenotype to an anti-inflammatory M2 phenotype, leading to inflammation reduction in Celiac disease [18].

Based on these results, in this study we decided to adopt a similar approach in IBD, examining the effect of CB2 receptor stimulation on macrophage polarization in pediatric patients with Crohn’s disease (CD) and ulcerative colitis (UC).

Our research group has already studied the role of CB2 receptor in IBD, demonstrating that a variant of the CB2 encoding gene (rs35761398-CB2 Q63R) may result in a less functional receptor isoform and is associated with an increased risk of developing inflammatory and autoimmune disorders [25]. In particular, we have already reported an association between this polymorphism and the susceptibility to pediatric IBD, particularly CD, and with a more severe phenotype in both UC and CD [14].

In this study, we observed a strong reduction in CB2 protein expression levels in IBD macrophages compared to CTR ones, suggesting its involvement in the pathogenesis of these pediatric pathologies.

In the present work, we investigated the macrophage population in IBD patients by assessing the protein expression levels of M1 markers (CCR7 and DMT1) and M2 markers (CD206 and pSTAT6). Specifically, we opted to evaluate CCR7 and CD206 as they are widely recognized as the most representative M1 and M2 markers, respectively [26,27,28]. Moreover, we analyzed the levels of pSTAT6, the phosphorylated and activated form of the STAT6 protein, which is an indicator of the transition from the M1 to the M2 macrophage phenotype [29,30].

As expected, considering the impaired inflammatory state characteristic of IBD, we found an increase in M1 macrophages, evidenced by elevated protein expression of CCR7 and DMT1. Conversely, there was a concurrent reduction in M2 macrophages, as indicated by lower expression levels of the M2-associated markers CD206 and pSTAT6. The inflammatory condition was also confirmed by an increased release of two potent pro-inflammatory cytokines, IL-6 and IL-23, both implicated in inflammation and immune system regulation [31,32,33,34,35]. Growing evidence highlights that IL-6, a pleiotropic pro-inflammatory cytokine produced by several cell types, particularly by intestinal macrophages, plays an essential role in maintaining metabolic, hepatic, and gastrointestinal homeostasis. It is known that IL-6 levels significantly rise during inflammation, driving its progression [31,36,37]. Interestingly, IL-6 family cytokines are now key therapeutic targets for several diseases, including inflammatory bowel disease (IBD) [31,36]. In line with this evidence, we found high levels of IL-6 in IBD, thus confirming once again the presence of an impaired inflammatory state in these diseases. We also evaluated IL-23 release, a cytokine produced by macrophages and dendritic and epithelial cells, known for its pivotal role in mucosal inflammation and which has emerged as a significant target for drug development [38,39]. It contributes to the development of colitis in various mouse models [36] and is implicated in the pathogenesis of both CD and UC [31]. Additional studies have highlighted an elevated expression of IL-23 in the mucosa of CD patients, as well as by CD14+ intestinal macrophages, which contribute to the maintenance of inflammation by infiltrating the inflamed intestine of CD patients [33]. Consistent with these findings, we observed an increase in IL-23 concentrations in IBD macrophages. Subsequently, we also examined IL-13 release. This anti-inflammatory cytokine has a pleiotropic function with effects on many cell types, including macrophages, and epithelial cells, and is thought to be involved in the pathological mechanisms underlying IBD [40]. We detected, in both diseases, a significant reduction in IL-13 levels, already known to exert an anti-inflammatory action through downregulation of the Th1–Th17 response.

Interestingly, we demonstrated that CB2 stimulation with its selective agonist, JWH-133, restored the inflammatory state, normally impaired in IBD, reducing both IL-6 and IL-23 levels, and increasing IL-13 release both in UC and CD macrophages, suggesting these cytokines as primary targets of CB2 in IBD.

Following treatment with JWH-133, we observed a phenotypic shift in macrophages toward the anti-inflammatory and immunosuppressive M2 phenotype. This was highlighted by evaluating both the expression of macrophage markers and by analyzing the cytokine profile. This result confirms not only the anti-inflammatory and immunomodulatory role of the CB2 receptor, but also supports recent studies on the role of CB2 in modulating macrophage polarization. Examples include research by Dalle et al. on muscle regeneration [41], Ryu et al. on pathways of inflammation and fibrosis in pancreatic diseases [42], and Olate-Briones et al. who demonstrated that the use of a natural herbal supplement in vivo promotes anti-inflammatory differentiation of M2 macrophages reducing the severity of colitis [43]. Therefore, to the best of our knowledge, our data suggest for the first time that CB2 stimulation, inducing the macrophage switch towards the anti-inflammatory M2 phenotype, could be an attractive and promising target for mitigating inflammation also in pediatric IBD.

Furthermore, it is well known that there is a strong correlation between iron and inflammation [44]. In our previous study, we already demonstrated that serum hepcidin is increased in IBD children with active disease and it is responsible for iron malabsorption [45]. Based on this evidence, our current study also aimed to investigate CB2 modulation of iron metabolism in IBD macrophages. Indeed, the increased levels of pro-inflammatory cytokines, mainly IL-6 [45], are responsible for the increased levels of hepcidin, which induces the degradation of FPN-1, the only known cell iron exporter, and, consequently, intracellular iron accumulation, contributing to worsening the inflammatory state [44]. According to this biological mechanism, together with the already mentioned increase in IL-6, we observed an increase in hepcidin levels, a reduction in FPN-1 protein expression levels, and an increase in intracellular iron concentration in IBD macrophages compared to those ones isolated by healthy donors. Moreover, we also found a significant increase in the iron importer DMT1. Collectively, these findings indicate an alteration of iron metabolism in IBD macrophages, which could be involved in the perpetuation of the inflammatory state in these diseases. Building on our previous studies on the role of CB2 in regulating iron metabolism [16,19], we investigated whether stimulating this receptor in macrophages isolated from pediatric IBD patients could modulate iron metabolism and counteract the characteristic inflammatory state of IBD.

Our findings demonstrated that the administration of a CB2 agonist effectively restored iron metabolism. Specifically, treatment with JWH133 resulted in a statistically significant reduction in hepcidin levels in both CD and UC macrophages, along with a marked increase in FPN-1 protein expression. To our knowledge, this is the first indication that CB2 stimulation may regulate iron metabolism through the hepcidin-FPN-1 signaling axis.

Interestingly, we also observed that CB2 agonist treatment influenced iron internalization by modulating DMT1 protein expression levels, leading to a notable reduction in both CD and UC macrophages. These findings suggest that CB2 may serve as a key modulator of iron metabolism in IBD macrophages, proposing a novel mechanism to counteract inflammation in pediatric IBD while also reinforcing our previous work.

Notably, we have demonstrated that CB2 can influence bone metabolism in pediatric IBD by indirectly regulating iron metabolism, thereby exerting anti-osteoporotic effects [16,19]. Furthermore, in a previous study, we provided evidence of CB2 receptor expression in the human intestine, highlighting its potential role in modulating inflammation and immune responses in pediatric IBD patients [46].

It is well-established that in IBD, the integrity of the intestinal epithelial barrier, which serves as the first line of defense, is strongly dysregulated, with a consequent increase in intestinal permeability which allows the entry of microorganisms and antigens into the lamina propria, leading to activation of the immune system and inducing the inflammatory response [47].

To assess the role of M1 macrophages derived from CD patients in compromising intestinal barrier integrity, and to further validate the protective potential of CB2 activation, we employed an ‘immunocompetent gut’ model [19,48], consisting of IBD-derived macrophages and the human epithelial cell line Caco-2, used as a representative model of the intestinal epithelial barrier. Our findings revealed elevated levels of the pro-inflammatory cytokines IL-6 and IL-23, along with a decreased number of viable Caco-2 cells when co-cultured with IBD macrophages compared to Caco-2 cells cultured alone, suggesting a role of M1 IBD macrophages in inducing mucosal barrier damage. Notably, stimulation of CB2 with JWH-133 ameliorated the barrier damage induced by M1 IBD macrophages, increasing the number of viable Caco-2 co-cultured with CD and UC macrophages and determining a reduction in pro-inflammatory cytokines compared to NT Caco2 co-cultured with IBD macrophages. These results suggest the role of CB2 in restoring the functionality of the mucosal barrier by modulating the IBD macrophage phenotype.

Although the small number of patients could be considered a limitation, it is important to note that this is a preclinical in vitro study conducted on primary cell cultures. A key strength of our study lies in the ability to obtain and analyze samples from pediatric subjects, which adds significant value given the challenges associated with research in this population. Additionally, while the use of peripheral blood-derived macrophages instead of intestinal tissue may be considered a limitation, it offers a minimally invasive approach to studying immune responses in IBD patients. Despite the technical challenges related to the low yields of intestinal macrophages from current protocols, we recognize the importance of tissue-derived cells for validating our findings. Therefore, we plan to complement our study with future analyses using macrophages isolated from intestinal biopsies. Nonetheless, the validation of results from macrophage cultures using an “immunocompetent gut” model underscores the utility of this culture system in exploring potential therapeutic strategies for pediatric IBD. Collectively, our data demonstrate that the CB2 receptor is implicated in inflammation in chronic IBD and highlights that the CB2 receptor could be a possible novel therapeutic target for IBD, through novel mechanisms of action, by modulating macrophage polarization and iron metabolism, and preventing mucosal barrier damage. Indeed, CB2 stimulation drives macrophage polarization toward the anti-inflammatory M2 phenotype and promotes iron release by cells, thus not only reducing inflammation but also helping to preserve epithelial barrier integrity. Further in vitro and in vivo investigations are warranted to confirm the potential anti-inflammatory role and the novel mechanism of action mediated by CB2 stimulation in containing and managing the impaired inflammatory state in IBD patients and its related side consequences. The potential clinical application of CB2 modulation in IBD remains a crucial area for future research. While selective CB2 agonists, such as JWH-133, have been extensively studied in preclinical in vitro and in vivo models to investigate CB2-mediated immunomodulation [7,49], their direct therapeutic use in IBD has yet to be established. Further research is needed to assess their safety and efficacy in vivo.

The development of novel CB2-targeting therapeutics, either alone or in combination with existing IBD treatments, could provide new strategies to modulate inflammation and restore intestinal barrier function. Future studies should explore the feasibility of CB2 modulation as an adjunctive or alternative approach to current IBD therapies.

## 4. Materials and Methods

### 4.1. Study Population

Our study population included a total of 50 pediatric subjects (age < 18 years), 17 of whom were affected by UC (mean age 10 years; range 1–18 years; female/male:10/7), 21 affected by CD (mean age 11.9 years; range 1–18 years; female/male: 10/11), all of whom were specifically selected for being wild-type for the Q63R variant [14], and 12 who were non-IBD controls (CTR; mean age 11.3 years; range 1–18 years; female/male: 5/7). All subjects were enrolled at the Department of Woman, Child and General and Specialist Surgery of the University of Campania Luigi Vanvitelli between 2021 and 2023. The CTR group consisted of subjects who underwent an esophagogastroscopy or ileo-colonoscopy to either exclude an organic disease or to remove juvenile polyps, and who did not present any signs of mucosal inflammation or disease. The patients with IBD were enrolled at diagnosis, before the start of medical treatment. The diagnoses of UC and CD were based on clinical, endoscopic, and histopathologic criteria [50].

Laboratory parameters and clinical and endoscopic characteristics of CTR and IBD subjects are reported in Table 1 and Supplementary Table S1, respectively.

All procedures conducted in this study are in agreement with the Helsinki Declaration of Principles, the Italian National Legislation, and the Ethics Committee of the University of Campania Luigi Vanvitelli, which formally approved the study (identification code Prot. 0013347/i del 29 April 2021). Written informed consent was obtained from parents, and approval was acquired from children before any proceedings.

### 4.2. Macrophage Cell Culture

Macrophages were obtained from mononuclear cells (PBMCs), isolated from peripheral blood by density gradient centrifugation (Ficoll 1.077 g/mL). The PBMCs were diluted 1× 10^6^ cells/mL in a complete culture medium consisting of α-minimal essential medium (α-MEM) (Lonza, Verviers, Belgium) supplemented with 10% fetal bovine serum (FBS) (Euroclone, Siziano, Italy), 100 IU/mL penicillin, and 100 g/mL streptomycin and L-glutamine (Gibco Limited, Uxbridge, UK), and then seeded in a 24-well plate. To obtain completely differentiated human macrophages, the PBMCs were cultured for 15 days in the presence of 25 ng/mL recombinant human macrophage colony-stimulating factor (rh-MCSF) (Peprotech, London, UK). Cells were incubated at 37 °C in a humidified atmosphere with 5% CO_2_, and the medium was replaced twice a week.

When macrophages were fully differentiated, cell culture supernatants were harvested to perform the Iron Assay and to analyze the release of pro- and anti-inflammatory cytokines by the enzyme-linked immunosorbent assay (ELISA). Cells were collected for protein extraction.

### 4.3. Caco-2 Cell Culture and Transwell System

Caco-2 cells, a human colorectal adenocarcinoma cell line bought from ATCC (accession number HTB-37), were cultured in minimum essential medium (MEM) supplemented with 10% FBS, 1% non-essential amino acids, and 1% antibiotics (100 U/mL penicillin and 100 μg/mL streptomycin), replacing culture medium twice a week. After reaching 80% confluence, Caco-2 was split, re-plated for expansion, and harvested until passage [18]. Therefore, it was possible to continue with Transwell experiments, performed as previously described [18].

Caco-2 cells were cultured for 14–20 days to confluency in the upper chamber of a 3 μm pore Transwell insert (Falcon, Franklin Lakes, NJ, USA). Transwell inserts with a differentiated Caco-2 monolayer were added into the Transwell plate pre-loaded with CD and UC macrophages, thus obtaining Caco-2 alone and Caco-2 plus CD or UC macrophages, treated or not with JWH-133 [100 nM] and AM630 [10 μM] for 48 h.

The supernatants of Caco-2 cells were collected to analyze IL-6 and IL-23 release and cells were used to perform cell viability assay.

### 4.4. Drugs and Treatments

Macrophages were treated for 48 h with JWH-133 [100 nM], a CB2 selective agonist, and with AM630 [10μM], a CB2 inverse agonist. Drugs were purchased as a powder from Tocris (Avonmouth, UK) and were dissolved in PBS containing dimethyl sulfoxide (DMSO). DMSO final concentration on cultures was 0.01%. Non-treated cultured macrophages were maintained in incubation media during the relative treatment time with or without a vehicle (DMSO 0.01%). The concentrations of the drugs were defined through concentration-response experiments and were represented by those ones which produced the strongest effects without modifying cell viability (Supplementary Figure S1).

### 4.5. Western Blot Analysis

Proteins were extracted from macrophage cultures using RIPA lysis and extraction buffer (Millipore, Milano, Italia), following the manufacturer’s instructions. Protein concentration was determined using the Bradford dye-binding assay (Bio-Rad, Hercules, CA, USA). To analyze the expression of proteins CB2, CCR7, DMT1, CD206, pSTAT6, and FPN-1 in the macrophages’ total lysates, Western blotting was performed, loading 15 μg of denatured proteins. Membranes were incubated overnight at 4 °C with these primary antibodies: rabbit polyclonal anti-CB2 (1:500 dilution; Elabscience, Houston, TX, USA) (molecular weight: 45 KDa), rabbit polyclonal anti-CCR7 (1:500 dilution; Elabscience, Houston, TX, USA) (molecular weight: 48 KDa), mouse monoclonal anti-DMT1 (1:100 dilution; Santa Cruz Biotechnology, Dallas, TX, USA) (molecular weight: 64 KDa), mouse monoclonal anti-CD206 (1:250 dilution; Santa Cruz Biotechnology, Dallas, TX, USA) (molecular weight: 170 KDa), rabbit polyclonal anti-pSTAT6 (1:500 dilution; Elabscience, Houston, TX, USA) (molecular weight: 110 KDa), rabbit polyclonal anti-FPN-1 (1:1000 dilution; Novus Biologicals LLC, Centennial, CO, USA) (molecular weight: 62.5 KDa), and then with the relative secondary antibody for 1 h. A mouse monoclonal anti-β-Actin antibody (1:100 dilution; Santa Cruz Biotechnology, Dallas, TX, USA) (molecular weight: 43 KDa) and a mouse monoclonal anti-GAPDH (1:2000 dilution; Thermo Fisher Scientific, Waltham, MA, USA) (molecular weight: 37 KDa) were used as housekeeping proteins. Immunoreactive bands were detected by chemiluminescence (Clarity and Clarity Max ECL Western Blotting Substrates, Bio-Rad Laboratories, Hercules, CA, USA), and the images were captured by C-DiGit blot scanner (LI-COR Biosciences, Lincoln, NE, USA) and by ChemiDoc MP Imaging System (Bio-Rad, Laboratories, Hercules, CA, USA). Image Studio Digits software (version 5.0.) and “Image Lab.Ink 6.1” software (Bio-Rad Laboratories, Hercules, CA, USA) were used for quantitative analysis of band intensity.

### 4.6. ELISA Assay

IL-6, IL-23, IL-13, and hepcidin concentration levels in the macrophage culture supernatants were determined by ELISA assays, through commercially available Human ELISA kits (Invitrogen by Thermo Fisher, Waltham, MA, USA). Standards and supernatants were loaded, in duplicate, to the wells of the microplate, already coated with monoclonal antibodies specific for the cytokines. After several washing steps, enzyme-linked polyclonal antibodies specific to each target were added to the wells. The reaction was developed by adding a substrate solution, and the resulting signal was measured as optical density at 450 nm using the Tecan Infinite M200 (Tecan Group Ltd., Männedorf, Switzerland) spectrophotometer. Cytokine concentrations (pg/mL) were determined against a standard concentration curve.

### 4.7. Iron Assay

Macrophage culture supernatants were collected after 48 h treatment, to evaluate the iron (III) concentration. The assay was performed using the Iron Assay Kit (Abcam, Cambridge, UK), and the macrophage supernatants were pipetted into the wells of a plate and incubated with an acidic buffer to allow iron release. Then, an iron probe was added at 25 °C for 60 min in the dark. Released iron reacted with the chromogen, producing a colorimetric product (593 nm), proportional to the iron concentration. The optical density was measured at 593 nm through the Tecan Infinite M200 (Tecan Group Ltd., Männedorf, Switzerland) spectrophotometer. Iron (II) and total iron (II + III) contents of the test samples (nmol/μL) were determined against a standard concentration curve. Iron (III) content can be calculated as: Iron (III) = Total Iron (II + III) − Iron (II).

### 4.8. Count and Viability Assay Kit

After 48 h of drug exposure, Caco-2 cells were isolated from the co-culture media in order to perform the count and viability assay, with the Muse cell analyzer machine using the Count & Viability Assay Kit (Merck KGaA, Darmstadt, Germany) The Muse Count & Viability reagent differentially stains viable and non-viable Caco-2 cells based on their permeability to the two DNA-binding dyes present in the reagent. A total of 450 μL of the Muse Count & Viability reagent was mixed with 50 μL of Caco-2 suspension (1 × 10^5^ cells/mL) and incubated for 5 min at room temperature. The results were analyzed with Muse 1.4 analysis software (Merck KGaA, Darmstadt, Germany).

### 4.9. Statistical Analysis

Statistical analyses were performed using GraphPad (Version 8.4.2; GraphPad software Inc., La Jolla, CA, USA). For statistical comparison, a *t*-test was used. For the statistical analysis of data obtained from ELISA, we compared the effects of the two treatments (JWH-133 and AM630) on Caco-2 macrophages with CD and UC against NT cells and Caco-2 alone. First, we performed the Shapiro-Wilk normality test to determine whether the sample distributions followed a normal distribution. Subsequently, a one-way ANOVA was conducted, followed by Tukey’s HSD post hoc analysis.

Data are expressed as mean ± SD, and statistical significance was set at *p* ≤ 0.05 (*).

## Figures and Tables

**Table 1:** This table shows the clinical characteristics of CTR and IBD subjects enrolled in the study (*n* = 50).

Laboratory Parameters	CTR	UC	CD
Median age, years (mean ± SD)	11.3	10	11.9
Sex (Female/Male)	5/7	10/7	10/11
Sideremia (µg/dL)	87.1 ± 33.9	63.4 ± 38.6	29.3 ± 19.7
Ferritin (ng/mL)	33.1 ± 20.5	22 ± 9.6	49.2 ± 56.4
Transferrin (mg/dL)	256.5 ± 14	291.3 ± 37.6	278 ± 61.9
C-Reactive Protein (mg/L)	0.084 ± 0.19	0.39 ± 0.76	2.9 ± 4.0
Hemoglobin (g/dL)	13.23 ± 1.00	12.4 ± 1.5	11.4 ± 1.3
Mean Corpuscular Volume fl	79.7 ± 3.6	79.3 ± 6.9	72.1 ± 8.8
Transferrin Saturation Index (%)	30.5 ± 10	14.3 ± 7.6	6.8 ± 4.4
Calprotectin (mg/Kg)	80.0 ± 32.7	506.8 ± 405.8	1425.9 ± 1002.9

## Data Availability

Our data are entered into a database and are available by agreement with the authors.

## Supplementary Materials

The following supporting information can be downloaded at: https://www.mdpi.com/article/10.3390/ijms26083720/s1.

## Abbreviations

The following abbreviations are used in this manuscript:

IBD	inflammatory bowel disease
CB2	cannabinoid receptor 2
UC	ulcerative colitis
CD	Crohn’s disease
PBMCs	mononuclear cells
α-MEM	α-minimal essential medium
FBS	fetal bovine serum
rh-MCSF	recombinant human macrophage colony-stimulating factor
ELISA	enzyme-linked immunosorbent assay
MEM	minimum essential medium
DMSO	dimethyl sulfoxide
WB	Western blot
