# Supplementary material for: Effects of CB2 Receptor Modulation on Macrophage Polarization in Pediatric Inflammatory Bowel Disease
Source: Int J Mol Sci. 2025 Apr 15;26(8):3720. doi: 10.3390/ijms26083720 (PMC12027514; doi:10.3390/ijms26083720)
Supplement: Supplementary file 1 [file ijms-26-03720-s001.zip › Supplementary Figure S1.pdf]

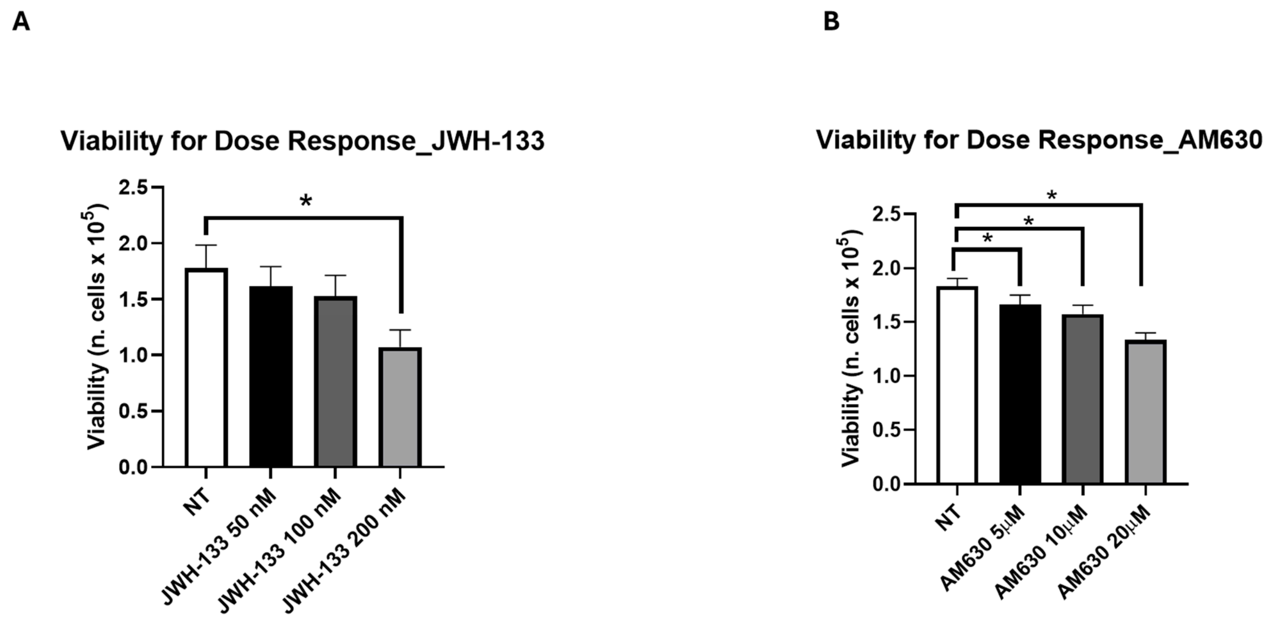

Supplementary Figure S1. Viability Assay on IBD macrophages treated with different concentration of JWH-133 and AM630. The viability of IBD macrophages was estimated by a cytofluorimetric assay after treatment with JWH-133 (50, 100, 200 nM)) and AM630 (5, 10, 20 μM). The histogram shows results as cell number × 10^5 and as mean ± SD of independent experiments on five different patients. * indicates $p \leq 0.05$ compared to NT.
