# Supplementary material for: Effects of CB2 Receptor Modulation on Macrophage Polarization in Pediatric Inflammatory Bowel Disease
Source: Int J Mol Sci. 2025 Apr 15;26(8):3720. doi: 10.3390/ijms26083720 (PMC12027514; doi:10.3390/ijms26083720)
Supplement: Supplementary file 1 [file ijms-26-03720-s001.zip › Supplementary Table S1.pdf]

| **Paris localization CD (*n*)** | **Total cohort *n* = 21** |
|---|---|
| **L1** | 1 |
| **L2** | 3 |
| **L3** | 7 |
| **L4a** | 1 |
| **L3L4a** | 6 |
| **L2L4a** | 1 |
| **L1L2** | 0 |
| **L1L4a** | 2 |
| **Extraintestinal Manifestation CD (n/tot cohort)** | 9/21 |
| **Paris localization** UC (***n***) | **Total cohort *n*=17** |
| **E1** | 7 |
| **E2** | 1 |
| **E3** | 1 |
| **E4** | 8 |
| **Extraintestinal Manifestation UC (*n*/tot cohort)** | 5/17 |

**Supplementary Table S1.** Clinical and endoscopic characteristics: Paris Classification and Extraintestinal Manifestation of IBD patients at diagnosis
